# Prolonged Lipid Accumulation in Cultured Primary Human Hepatocytes Rather Leads to ER Stress than Oxidative Stress

**DOI:** 10.3390/ijms21197097

**Published:** 2020-09-26

**Authors:** Christiane Rennert, Theresa Heil, Gerda Schicht, Anna Stilkerich, Lena Seidemann, Victoria Kegel-Hübner, Daniel Seehofer, Georg Damm

**Affiliations:** 1 Department of Hepatobiliary Surgery and Visceral Transplantation, University Hospital, Leipzig University, 04103 Leipzig, Germany; christiane.rennert2@medizin.uni-leipzig.de (C.R.); gerda.schicht@sikt.uni-leipzig.de (G.S.); anna.stilkerich@medizin.uni-leipzig.de (A.S.); lena.seidemann@medizin.uni-leipzig.de (L.S.); victoria.kegel@yahoo.de (V.K.-H.); daniel.seehofer@medizin.uni-leipzig.de (D.S.); 2 Saxonian Incubator for Clinical Translation (SIKT), Leipzig University, 04103 Leipzig, Germany; 3 Department of General-, Visceral- and Transplantation Surgery, Charité University Medicine Berlin, 13353 Berlin, Germany; theresa.heil@outlook.de

**Keywords:** liver, primary human hepatocytes, steatosis, NAFLD, NASH, ER stress, oxidative stress

## Abstract

Overweight has become a major health care problem in Western societies and is accompanied by an increasing incidence and prevalence of non-alcoholic fatty liver disease (NAFLD). The progression from NAFLD to non-alcoholic steatohepatitis (NASH) marks a crucial tipping point in the progression of severe and irreversible liver diseases. This study aims to gain further insight into the molecular processes leading to the evolution from steatosis to steatohepatitis. Steatosis was induced in cultures of primary human hepatocytes by continuous five-day exposure to free fatty acids (FFAs). The kinetics of lipid accumulation, lipotoxicity, and oxidative stress were measured. Additionally, ER stress was evaluated by analyzing the protein expression profiles of its key players: PERK, IRE1a, and ATF6a. Our data revealed that hepatocytes are capable of storing enormous amounts of lipids without showing signs of lipotoxicity. Prolonged lipid accumulation did not create an imbalance in hepatocyte redox homeostasis or a reduction in antioxidative capacity. However, we observed an FFA-dependent increase in ER stress, revealing thresholds for triggering the activation of pathways associated with lipid stress, inhibition of protein translation, and apoptosis. Our study clearly showed that even severe lipid accumulation can be attenuated by cellular defenses, but regenerative capacities may be reduced.

## 1. Introduction

With the growing prevalence of obesity and its associated hepatic disorder, non-alcoholic fatty liver disease (NAFLD), obesity has become the main cause of chronic liver injury in developed countries [1]. Hepatic steatosis, the first stage of NAFLD, is characterized by the intracellular accumulation of lipid droplets in more than 5% of hepatocytes. When simple steatosis is accompanied by inflammation and cellular injury, the next stage, non-alcoholic steatohepatitis (NASH), is reached [2]. The progression to NASH onset marks a crucial point where the risk of further progression to liver cirrhosis and hepatocellular carcinoma (HCC) is not only increased, but also markedly accelerated. As many as 25% of NASH patients develop cirrhosis and/or HCC [3], and a significantly higher number of NASH-associated HCC cases are found in people with noncirrhotic livers, emphasizing the malignant potential of this condition compared to other underlying causes of liver injury [4].

The underlying molecular mechanisms that induce the progression from steatosis to steatohepatitis have not been fully elucidated. According to the “multiple-hit hypothesis”, hepatic lipid accumulation represents one of multiple concurrent insults, such as insulin resistance, mitochondrial dysfunction, and oxidative and endoplasmic reticulum (ER) stress, eventually leading to cellular damage and apoptosis [5,6].

Reactive oxygen species (ROS) are byproducts of mitochondrial oxidative processes, such as free fatty acid (FFA) β-oxidation. In a physiological state, ROS are detoxified by antioxidants, including glutathione (GSH) [7]. In steatotic cells, β-oxidation is presumably increased to such a high level that the accumulating ROS overwhelm the antioxidative capacities of the cell, fueling the oxidative damage of cellular structures and DNA [8,9,10,11,12]. Furthermore, ROS promote fibrogenesis by activating hepatic stellate cell (HSC) proliferation and collagen synthesis [13].

Experimental studies have shown that ER stress is another component involved in the development of steatohepatitis and liver fibrosis [14,15]. It has been established that the accumulation of misfolded proteins leads to the activation of three ER stress sensors, the mediators of the so-called unfolded protein response (UPR): Inositol-requiring enzyme 1a (IRE1a), PKR (protein kinase R)-like ER kinase (PERK), and activating transcription factor 6a (ATF6a). In their inactive state, these three transmembrane proteins are bound to the intracellular chaperone called binding immunoglobulin protein (BiP), which dissociates upon binding to misfolded proteins, permitting intracellular signal transmission [16]. The downstream signaling pathways of activated IRE1a, PERK, and ATF6a are thought to restore homeostasis in protein biosynthesis by enhancing cellular protein-folding capacities, increasing ER-associated protein degradation, upregulating autophagy, and inhibiting translation [17]. When the UPR is chronically activated and regeneration fails, the signals shift to the pathway that initiates apoptosis [18]. Liver biopsies of NASH patients feature an altered expression of ER stress-related genes with an upregulation of apoptosis- and autophagy-associated genes [19]. There is increasing evidence that an elevated supply of saturated FFAs, such as palmitate, triggers the UPR in an alternative way because of an expanded integration of phospholipids into the ER membrane [20,21,22]. Saturated FFAs may also contribute to the proapoptotic shift, as indicated by palmitate-treated hepatocytes inhibiting autophagosome-lysosome fusion [23,24].

This study aims to gain further insight into the molecular processes leading to the evolution from steatosis to steatohepatitis. Steatosis was induced in primary human hepatocytes (PHHs) upon exposure to palmitate and oleate. The levels of oxidative stress were measured directly and indirectly, and the protein expression profiles of the three major UPR signals (PERK, IRE1a, and ATF6a) were analyzed. Our results suggest that, although both oxidative and ER stress are induced in steatotic hepatocytes, a leading role in the progression to inflammation and apoptosis can be ascribed to the translation-inhibiting properties of the UPR.

## 2. Results

### 2.1. The Initial Lipid Content Influences Lipid Accumulation In Vitro

**Figure 1:**
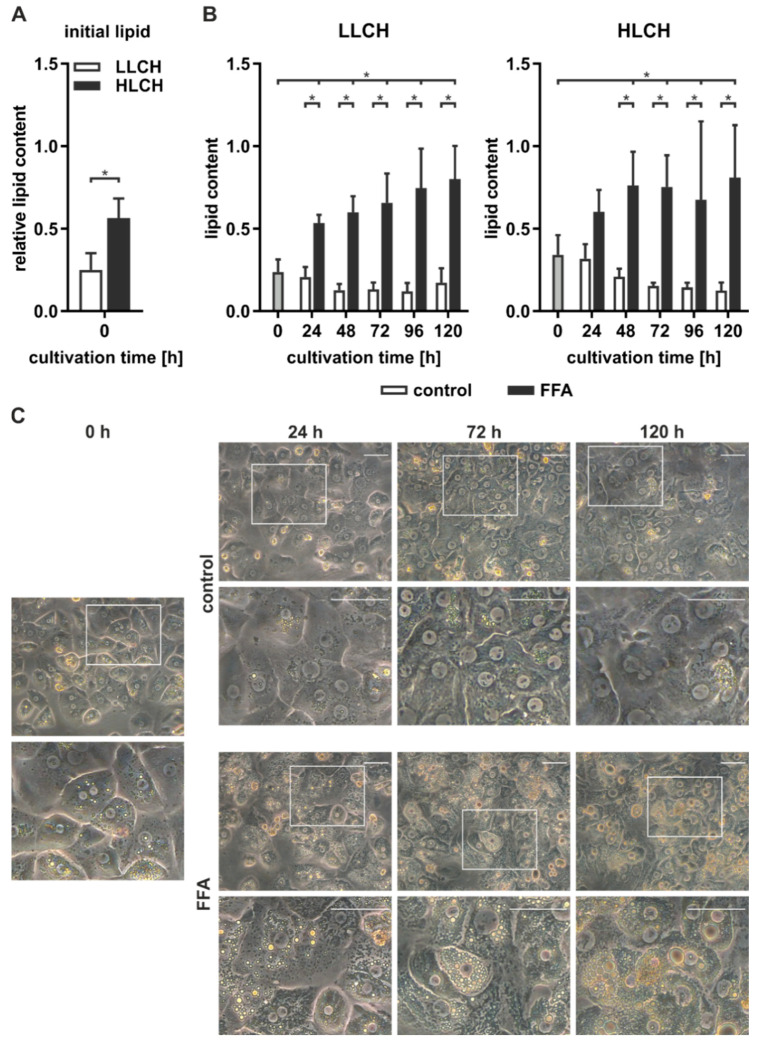
Increased lipid level after induction of in vitro steatosis. Primary human hepatocytes (PHHs) were continuously treated with control medium or 0.6 mM free fatty acids (FFA) every 24 h over five days. (**A**,**B**) Lipid level was quantified with Oil Red O assay. (**A**) Initial lipid content of the PHHs relative to sulforhodamine B (SRB). Depending on the initial lipid content, hepatocytes from donors 1–3 were classified in the low-lipid-containing hepatocyte (LLCH) group and those from donors 4–6 in the high-lipid-containing hepatocyte (HLCH) group. Data are shown as the means ± SD, *n* = 3, unpaired t-test, *p* < 0.05. (**B**) Quantification of the lipid content in the LLCH and HLCH cultures. Data are shown as the means ± SD, *n* = 3, two-way ANOVA, and post hoc Dunnett or Bonferroni analysis. A *p* value of 0.05 or less (*) was considered significant. For the details on the statistical evaluation, see Supplementary Table S1A. (**C**) Microscopic evaluation of the lipid accumulation in representative PHH cultures of donor 1 (magnification 32×). The white box indicates the enlarged section below. The scale bar is 50 µm.

We isolated PHHs from six human liver tissue samples (Table 1, donors 1–6) and determined the initial lipid content of the PHHs, remeasuring it every 24 h using microscopy and the Oil Red O assay. The PHH samples were categorized according to the initial relative lipid content and allocated to the low-lipid-containing hepatocyte group (LLCH) or the high-lipid-containing hepatocyte group (HLCH) (Figure 1A).

The seeded PHHs were treated with 0.6 mM FFA every 24 h for five days to induce steatosis in vitro. Microscopic observation of PHH cultures from donors 1–6 continuously treated with FFA showed a time-dependent accumulation of neutral lipids (Figure 1C). During the cultivation and FFA treatment, the amount, size, and location of lipid droplets varied among different hepatocytes. Many PHHs showed a movement of smaller lipid droplets towards the plasma membrane, while larger lipid droplets accumulated around the ER. The control cultures showed low lipid levels that were comparable to their initial levels.

Quantification of the lipid accumulation in the PHH cultures of cells from donors 1–6 showed different dynamics and capacities for lipid storage in the LLCH and HLCH (Figure 1B). PHHs in both groups exhibited a significantly steep increase in stored lipids during the first 24 h. Then, the LLCH showed a lower but continuous and highly significant lipid accumulation over the cultivation period. In contrast, the lipid storage in the HLCH linearly increased for additional 24 h, reaching significant saturation after 48 h. In both control groups, the lipid content decreased after 24 h to a lower level in a time-dependent manner, suggesting homeostasis was reached at lower equilibrium concentrations of lipids. The maximum value for lipid storage after five days was comparable in both groups with a 3.4-fold and a 2.4-fold significant increase in the LLCH and HLCH groups, respectively, compared to their respective initial values.

### 2.2. Continuous Lipid Accumulation Did not Lead to Cell Death

**Figure 2:**
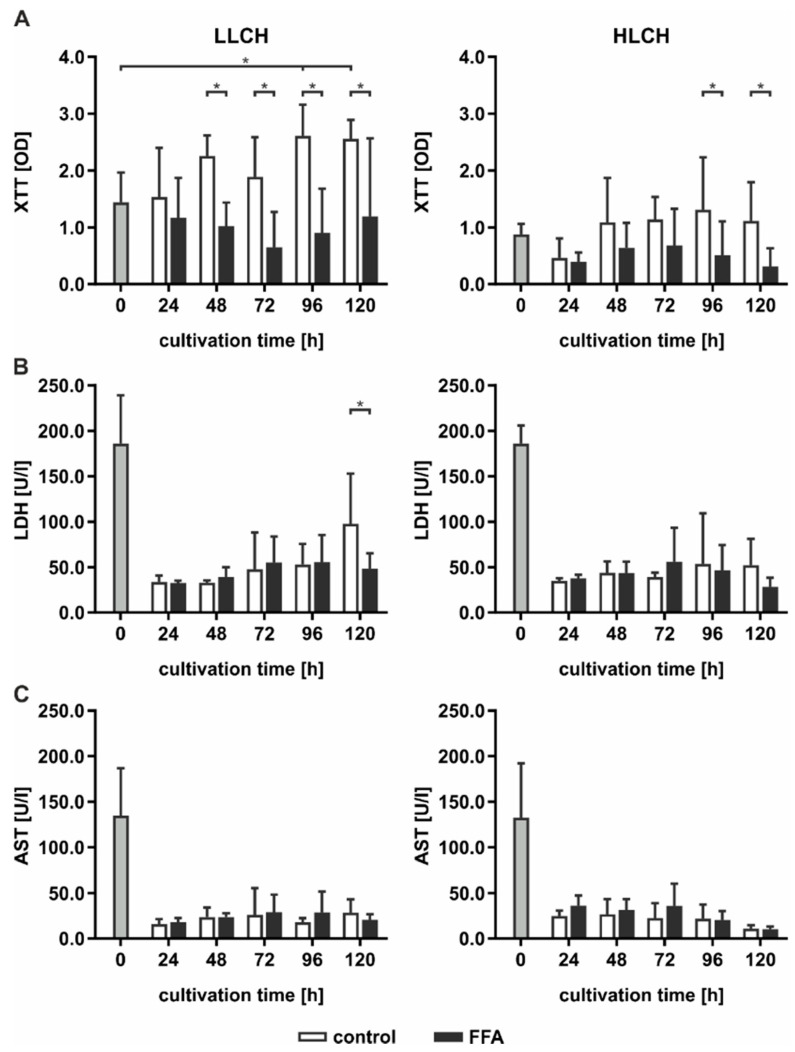
Evaluation of lipid-induced cell death in the LLCH (donors 1–3) and the HLCH (donors 4–6) according to the continuous lipid accumulation for five days. Lipotoxic effects were investigated by measuring cell activity levels using (**A**) XTT (2,3-bis-(2-methoxy-4-nitro-5-sulfophenyl)-2H-tetrazolium-5-carboxanilid) assays, and alterations to membrane integrity using extracellular (**B**) LDH (lactate dehydrogenase) and (**C**) AST (aspartate transaminase) enzyme activities. Data are shown as the means ± SD, *n* = 3, two-way ANOVA, and post hoc Dunnett or Bonferroni analysis. A *p* value of 0.05 or less (*) was considered significant. Selected comparisons are shown; for details on the statistical evaluation, see Table S1B.

Continuous lipid accumulation in vivo leads to fatty liver, whereas excessive lipid storage is linked to pathological tissue transformation and hepatocyte loss. Therefore, we sought to determine whether prolonged treatment of PHHs with FFA increases cell death rates. Pathological effects were evaluated by the measurement of cell activity using XTT (2,3-bis-(2-methoxy-4-nitro-5-sulfophenyl)-2H-tetrazolium-5-carboxanilid) assays, and membrane integrity was evaluated by measurements of LDH (lactate dehydrogenase) and AST (aspartate transaminase) release (Figure 2).

In general, cell activity levels were lower in the HCLH than in the LLCH group (Figure 2A). In the LLCH, the cell activity was significantly increased during the culture time of five days compared to that of the control group, reaching a plateau at day 4 with 2-fold higher values than the initial levels. In contrast, continuous FFA treatment led to a slight decrease in cell activity with varying and significantly lower values of cells treated with FFAs than were found for those not treated with FFAs. In the HLCH, a decrease in cell activity was observed in the first 24 h independent of FFA treatment. Between 24 and 48 h, the cell activity of the control group stabilized and reached the initial value and one half of the initial values in the FFA-treated PHHs. Prolonged FFA treatment led to significantly lower cell activity in the FFA-treated PHHs than in the control group.

The initially high LDH and AST values showing substantial deviation among donors reflected the stress of PHH isolation. LDH (Figure 2B) and AST (Figure 2C) activity levels significantly decreased after 24 h of cultivation, independent of FFA treatment, to a low basal level. Continuous FFA treatment did not lead to an increase in enzyme activity, and therefore, no signs of altered membrane integrity were observed.

Altogether, we did not observed signs of lipotoxicity as a result of the continuous in vitro lipid accumulation for five days.

### 2.3. Prolonged Lipid Accumulation Did not Lead to an Increase in ROS Level

**Figure 3:**
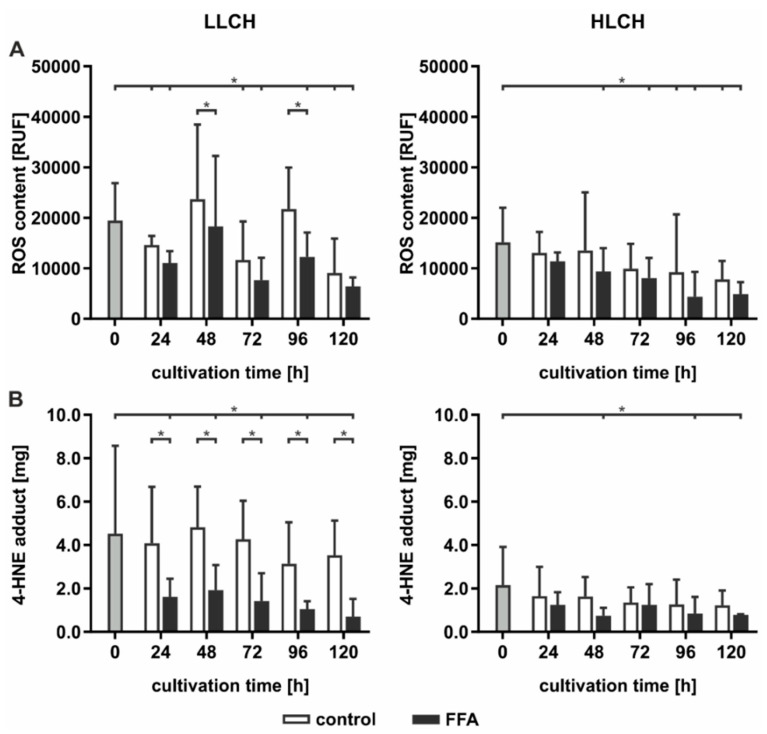
Measurement of reactive oxygen species (ROS) formation and 4-HNE-protein adducts in control and free fatty acid (FFA)-treated LLCH (donors 1–3) and HLCH (donors 4–6). (**A**) ROS formation was directly measured using DCF (dichlorodihydrofluorescein) assay. (**B**) 4-HNE-protein adducts were quantified by ELISA. Data are shown as the means ± SD, *n* = 3, two-way ANOVA, and post hoc Dunnett or Bonferroni analysis. A *p* value of 0.05 or less (*) was considered significant. For the details on the statistical evaluation, see Table S1C.

High hepatic lipid content is associated with increased oxidative damage linked to increased ROS formation generated by FFA-stimulated mitochondrial ß-oxidation. Therefore, we investigated whether continuous lipid accumulation leads to an increase in ROS formation. ROS were measured directly using DCF (dichlorodihydrofluorescein) assays and indirectly by investigation of the level of lipid peroxidation and availability of intracellular antioxidants. Lipid peroxidation leads to the formation of electrophilic 4-hydroxynonenal (4-HNE), which can be quantified as 4-HNE-protein adducts by ELISA. The influence on the intracellular antioxidant status was quantified by GSH availability and formation of oxidized GSH, that is, the GSSG level. The LLCH and HLCH showed significant time-dependent decreases in ROS with tendentially lower ROS values after FFA treatment than were shown in the control group (Figure 3A). These data were confirmed by the level of lipid peroxidation. Additionally, the amount of 4-HNE-protein adducts was approximately twice as high in LLCH as in HLCH group with significant lower values in the FFA-treated PHH (Figure 3B).

**Figure 4:**
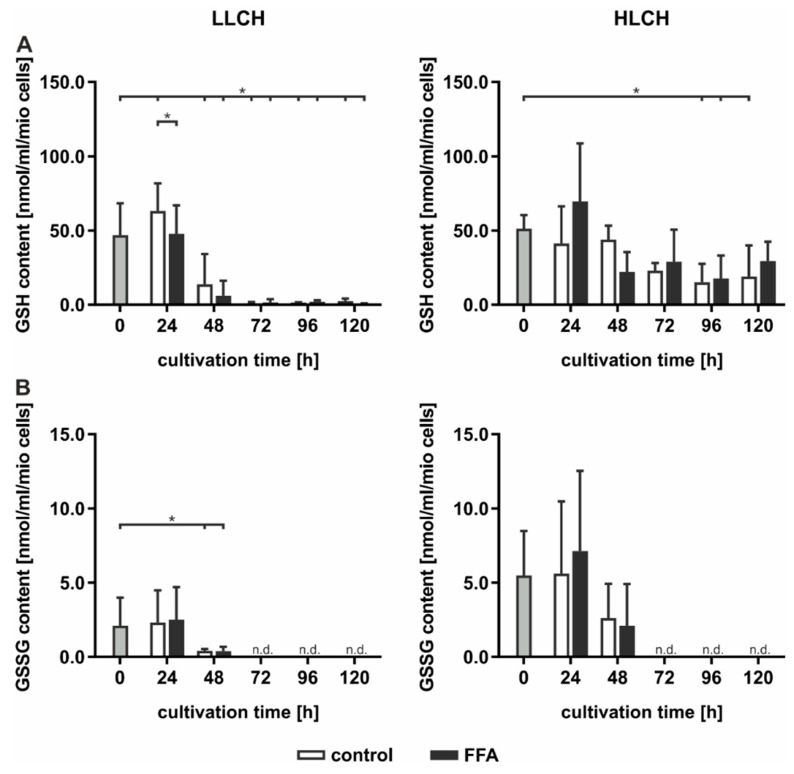
Measurement of the glutathion (GSH) and glutathione disulfide (GSSG) concentrations in the LLCH (donors 1–3) and HLCH (donors 4–6) depending on free fatty acid (FFA) treatment. (**A**) GSH and (**B**) GSSG (which was not detectable (n.d.) from 72 to 120 h) were quantified using Ellmann’s assay. Data are shown as the means ± SD, *n* = 3, two-way ANOVA, and post hoc Dunnett or Bonferroni analysis. A *p* value of 0.05 or less (*) was considered significant. For the details on the statistical evaluation, see Table S1D.

GSH is a strong endogenous antioxidative compound that detoxifies ROS by oxidizing GSH to generate GSSG. GSH and GSSG were quantified using Ellman’s assay (Figure 4). The initial basal GSH concentration of approximately 50 nmol/mL GSH/1 mio PHH was equal in both groups and was obtained for the first 24 h, while the GSH levels significantly decreased after the first 24 h of treatment (Figure 4A). The decrease in the GSH content in the FFA-treated and control cells in the HLCH group reached a low level of approximately 20 nmol/mL/1 mio PHH after 96 h. In the LLCH, the GSH stores of both the control and FFA-treated PHHs were depleted after 48 h (Figure 4A). Through the first 24 h, the GSSG concentration of the HLCH was more than twice as high (approximately 5 nmol/mL/1 mio PHH) as that of the LLCH (approximately 2 nmol/mL/1 mio PHH), suggesting a higher oxidative stress level in the HLCH (Figure 4B). With ongoing cultivation, after 24 h, the GSSG concentrations decreased, similar to the GSH concentrations, until GSSG was no longer detectable, after 72 h.

Altogether, we were not able to detect elevated ROS levels in steatotic hepatocytes as described in many in vivo studies. However, the initial GSSG concentration was higher in the HLCH than it was in the LLCH, which suggests increased oxidative stress in steatotic PHHs in vivo. The additional in vitro lipid accumulation was not accompanied by elevated ROS levels. The results suggest that a certain lipid load protects against ROS damage.

### 2.4. Higher Lipid Content Protects Against Oxidative Stress

To test the hypothesis that steatotic hepatocytes are more robust to oxidative damage, we induced oxidative stress in cultures of steatotic hepatocytes using menadione. Menadione induces the generation of ROS via redox cycling, leading to GSH depletion and cell death [25]. PHHs were treated without and with 0.6 mM FFA for 72 h, as described above for the lipid accumulation assay (Supplementary Figure S1), and for an additional 24 h with menadione at a concentration in the range of 1–10 µM.

**Figure 5:**
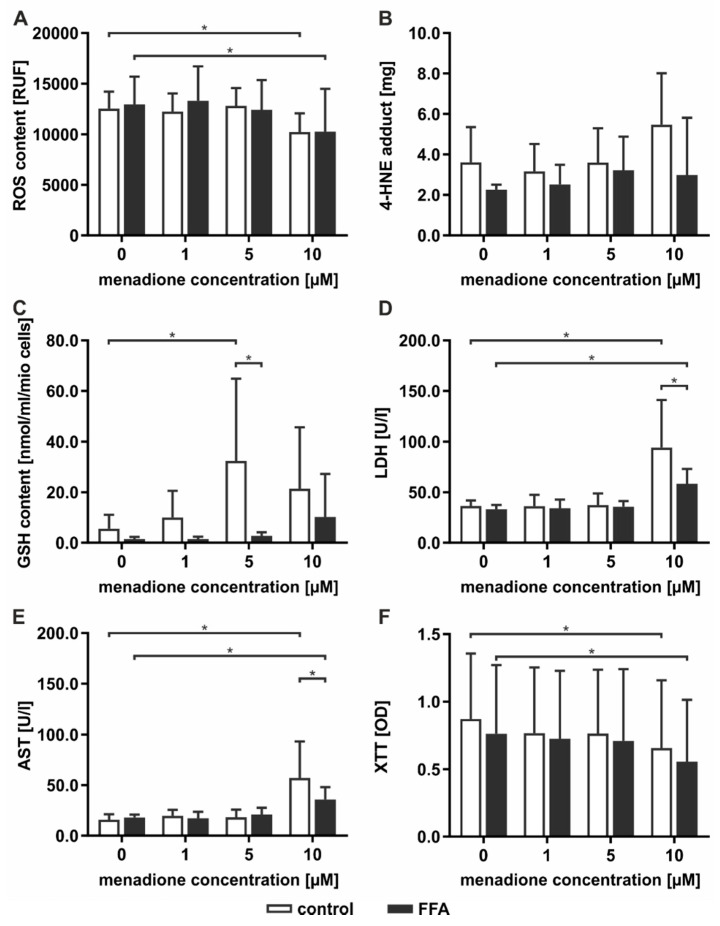
Induction of the oxidative stress by 0–10 µM menadione in the control and steatotic PHHs. (**A**–**C**) The oxidative stress level was evaluated (**A**) directly by measuring ROS levels and (**B**,**C**) indirectly by quantifying (**B**) the level of 4-HNE-protein adducts and (**C**) GSH levels. (**D**–**F**) The toxicity induced by menadione was investigated by measuring the alterations in membrane integrity as determined by extracellular (**D**) LDH and (**E**) AST enzyme activity and (**F**) cell activity by XTT assay. Data are shown as the means ± SD, *n* = 4 (donors 7–10), two-way ANOVA, and post hoc Dunnett or Bonferroni analysis. A *p* value of 0.05 or less (*) was considered significant. For the details on the statistical evaluation, see Table S1E.

Control and FFA-treated PHHs showed comparable ROS levels when incubated with the vehicle control and 1 and 5 µM menadione, while the 10 µM menadione treatment led to a significant decrease in the ROS levels in both groups (Figure 5A). Measurement of 4-HNE-protein adducts revealed a menadione concentration-dependent increase in lipid peroxidation, which was more pronounced in the control group than in the FFA-treated PHH (Figure 5B). The initial GSH levels were lower than 5 nmol/mL/mio cells, as shown for the PHHs cultured for 72 h (Figure 4A). However, the additional treatment with menadione increased the GSH concentration (Figure 5C). This increase was more pronounced in the control PHHs, in which it was evident at lower menadione concentrations than it was in the steatotic PHHs. Measurements of membrane integrity showed a toxic effect of incubations with 10 µM menadione, as indicated by the detection of LDH and AST metabolic activity in both the control and FFA-treated PHHs, but the toxicity was significantly lower in the steatotic PHHs (Figure 5D,E). The cell activity, measured by XTT assay, confirmed the toxicity of 10 µM menadione (Figure 5F).

Taken together, the data show that the incubation of PHHs cultured for 72 h without and with the addition of FFA and with 10 µM menadione led to oxidative stress in the PHHs. The oxidative damage was more pronounced in the control PHHs, while the steatotic PHHs showed less sensitivity towards ROS.

### 2.5. Prolonged Lipid Accumulation Led to ER Stress and Decreased Protein Synthesis

**Figure 6:**
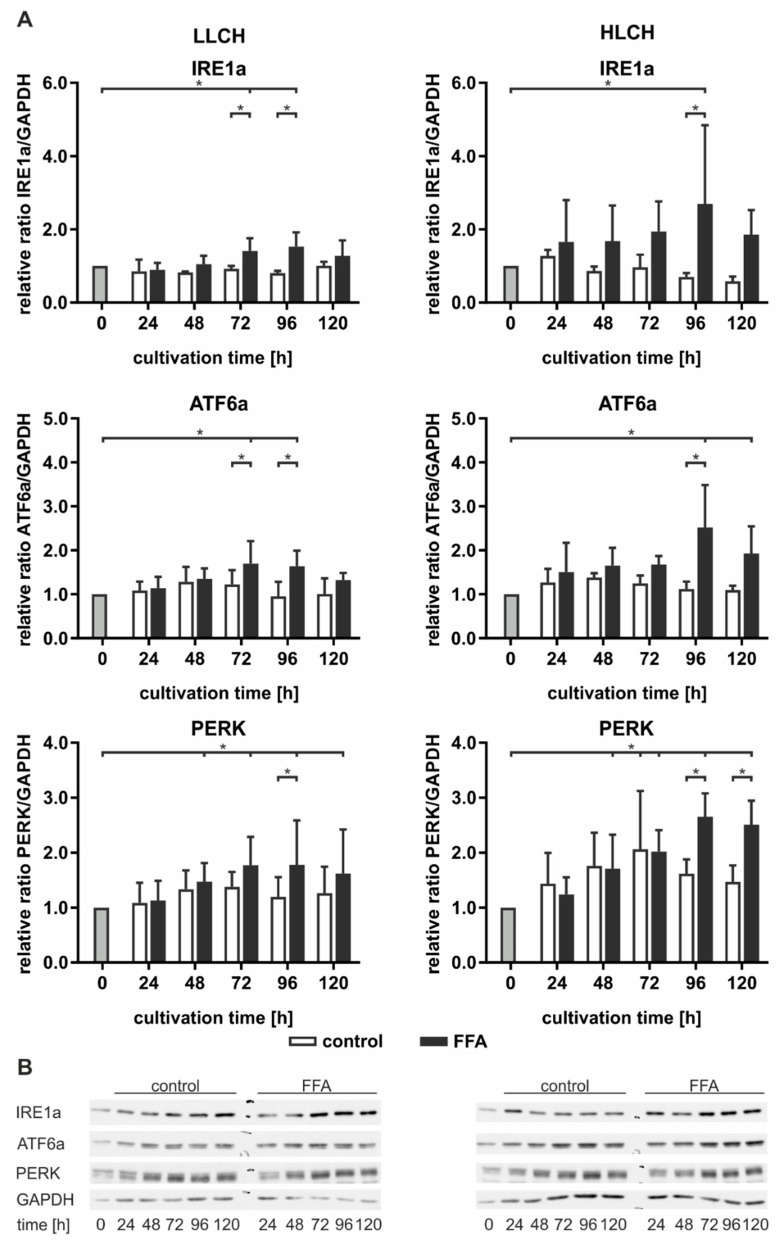
Prolonged lipid accumulation over 5 d led to lipid level-dependent endoplasmic reticulum (ER) stress and activation of the unfolded protein response (UPR). The UPR signaling targets IRE1a, ATF6a, and PERK were quantified by Western blot analysis in the LLCH (donors 1–3) and HLCH (donors 4–6). GAPDH was used as a reference protein. (**A**) Densitometrically analysis of Western blots. Data are shown as the means ± SD, *n* = 3, two-way ANOVA, and post hoc Dunnett or Bonferroni analysis. A *p* value of 0.05 or less (*) was considered significant. For the details on the statistical evaluation, see Table S1F. (**B**) Representative Western blots from donor 1 and 5 show IRE1a (130 kDa), ATF6a (90 kDa), PERK (140 kDa), and GAPDH (36 kDa) signals in the control and FFA-treated PHHs. For further Western blots, see Figures S2 and S3.

ER stress results in the activation of the UPR mediated by the signaling pathways of IRE1a, PERK, and ATF6a. These pathways and their downstream targets inhibit protein translation (PERK) and induce autophagy and protein turnover (IRE1a) and lipid metabolism (ATF6a) [16]. When the mechanisms fail to reduce ER stress, prolonged activation leads to the induction of apoptosis (PERK). As prolonged lipid accumulation leads to an induction of ER stress, we investigated the effect of various levels of stored lipids on the targets of the UPR pathways. Therefore, the LLCH and HLCH were incubated with 0.6 mM FFA as described above, and the protein samples were analyzed by Western blotting (Figure 6).

In general, we observed a slight time-dependent increase in ATF6a and PERK expression in the control LLCH and HLCH at early time points. These values decreased over the cultivation time, suggesting that ER stress was related to the initial lipid load and decreased as a result of time-dependent lipid degradation in the control PHHs. In particular, PERK expression was strongly correlated with the lipid levels in the PHHs. In contrast, the time-dependent course of IRE1a expression was stable in the control LLCH and decreased in a time-dependent manner in the HLCH (Figure 6).

In the LLCH, the lipid accumulation through 48 h did not lead to changes in UPR signaling proteins. Additional lipid storage led to a significant time- and lipid level-dependent increase in IRE1a, ATF6a, and PERK expression. The data indicate that prolonged lipid accumulation in the LLCH led to activation of autophagy (IRE1a) and lipid metabolism (ATF6a), but progression to apoptosis (PERK) was not clearly detected (Figure 6).

In contrast, we observed an immediate increase in IRE1a and ATF6a in the FFA-incubated HLCH after 24 h, which remained at this level through 72 h independent of further lipid accumulation. In this time span, the lipid accumulation was tolerated, as PERK expression was not increased in the FFA-treated HLCH compared to the level in the control HLCH. Further 24 h lipid storage (96 h) led to a significant peak in IRE1a and ATF6a expression accompanied by a significant increase in PERK expression. Furthermore, at 96 h, all these protein levels were significantly elevated in the FFA-treated PHHs compared to those in the control group. The IRE1a and ATF6a levels decreased after 120 h of lipid accumulation, while the PERK levels remained constant (Figure 6).

Taken together, the data suggest that lipid accumulation for 72 h in the HLCH led to ongoing activation of autophagy, protein metabolism (IRE1a), and lipid metabolism (ATF6a). Further lipid accumulation exceeded the threshold favoring the inhibition of translation and induction of apoptosis (PERK) over the induction of repair mechanisms (IRE1a, ATF6a), leading to a persistent high PERK level and reduction of IRF1a and ATF6a levels.

**Figure 7:**
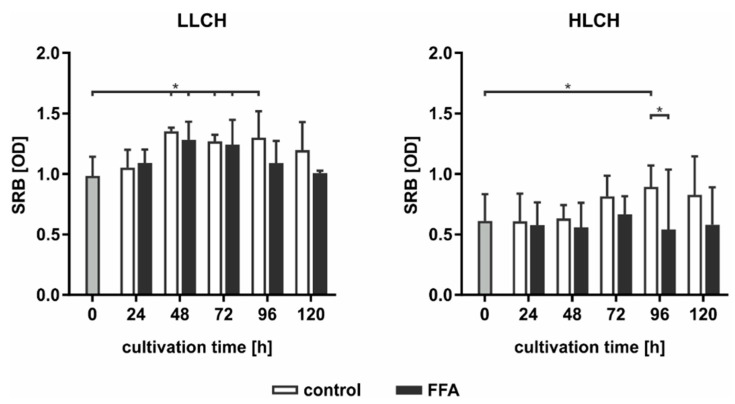
PERK activation leads to the inhibition of protein expression on the cell surface. In the LLCH (donors 1–3) and HLCH (donors 4–6), the amount of surface proteins was measured using SRB staining. Data are shown as the means ± SD, *n* = 3, two-way ANOVA, and post hoc Dunnett or Bonferroni analysis. A *p* value of 0.05 or less (*) was considered significant. For the details on the statistical evaluation, see Table S1G.

As PERK activation leads to the inhibition of protein translation, we used SRB staining to investigate the total expression of membrane proteins dependent on the lipid load. In general, we found an approximately 2-fold higher level in the initial amount of surface protein on the LLCH compared to the amount on the HLCH (Figure 7). In LLCH, the surface protein amount increased during the first 48 h of cultivation with increasing lipid load. Further lipid accumulation led to a decrease in the surface protein level in the FFA-treated LLCH, while the surface protein level remained constant in the control LLCH. In contrast, in the cultures of the HLCH, the surface protein level remained constant for the first 48 h independent of lipid load. Further cultivation of the HLCH control PHHs led to a significant increase in the amount of surface protein, while further culturing in the presence of FFA sustained the HLCH at the low basal level of surface protein expression (Figure 7).

Taken together, the data suggest that the first 48 h of primary cell culture was dominated by regenerative processes that restored surface protein expression levels after proteolytic damage incurred during the cell isolation process. Then, this protein amount remained constant (LLCH control), while a higher lipid load (LLCH FFA) resulted in a decrease in the surface protein level or the inhibition of the initial regeneration of these proteins (HLCH). The decrease in the lipid level by a specific amount in the HLCH control cells enabled the restoration of their surface proteins. Therefore, the lipid load negatively influences the ability of the hepatocytes to undergo regenerative processes.

## 3. Discussion

Advanced steatosis in vivo is known to lead to severe lipid accumulation that is linked to oxidative and ER stress. The activation of both stress pathways leads to increased cellular regenerative capacities that reduce the cause of cell stress. Therefore, oxidative stress leads to the induction of antioxidative activity, resulting in an enhanced availability of the endogenous antioxidative peptide GSH. However, ER stress also activates the unfolded protein response (UPR), leading to higher protein and lipid turnover to eliminate and recycle misfolded proteins and lipids. If both pathways fail to reduce the stress level, then apoptotic pathways are activated. The persistent stress of cells results in cell death, as observed in the progression from steatosis to steatohepatitis.

Therefore, this study aimed to gain further insight into the molecular processes leading to the evolution from steatosis to steatohepatitis in humans.

### 3.1. The Progress of Lipid Accumulation Was Based on the Initial Lipid Level

Persistent high blood concentrations of free fatty acids (FFAs) increase lipid accumulation in the form of triacylglycerides (TAGs) in hepatocytes [26]. The investigation of lipid accumulation after FFA treatment in different batches of PHH showed two types of dynamics that were dependent on the initial lipid level. PHHs with low lipid content (LLCH) showed a sharp increase in lipid accumulation in the first 24 h of FFA treatment. Then, the rate of lipid droplet formation was linear for four days. In contrast, PHHs with high lipid content (HLCH) showed a high level of accumulated lipids during the first 48 h, at which point, saturation was reached and the level remained constant for four days. However, the maximum lipid uptake capacity was similar for the LLCH and HLCH.

The different dynamics of in vitro lipid accumulation are associated with the physiology of the liver before hepatocyte isolation. Since the HLCH reflects the NAFLD condition, adapted expression of lipid transporters in hepatocyte membranes may be the basis of the higher lipid uptake rate. CD36 was shown to be abnormally increased under the NAFLD condition [27], while under a healthy condition, CD36 plays a minor role in hepatic FFA uptake [28]. Further metabolic adaptations such as elevated metabolism and VLDL excretion [29] can balance the hepatic lipid load, which is reflected in the decelerated lipid droplet formation after 24 h in the LLCH. However, after a certain time, the maximum lipid load is reached. To determine whether this maximum lipid amount reflects maximum storage capacity or an equilibrium of uptake, metabolism, and excretion, further experiments are needed.

### 3.2. Severe Lipid Accumulation Did not Lead to Cell Death

Hepatic TAG accumulation is considered a nontoxic mechanism of lipid storage in the liver [5]. Our experimental data on cell activity and membrane integrity confirmed that prolonged lipid accumulation, even that resulting in a severe lipid load, did not lead to lipotoxic effects. In various experimental settings, the exposure of cultured cells to unsaturated FFAs resulted in significantly increased TAG content without a decrease in cell viability [30]. We used a 2:1 ratio of oleic acid to palmitic acid. This FFA mixture is associated with minor toxic and apoptotic effects, thus representing a cellular model of steatosis in which unsaturated FFAs serve a protective function against lipotoxicity, mimicking benign chronic steatosis [31]. Our data confirmed that using this mixture of FFAs led to a lipid load that was well tolerated by the PHHs.

### 3.3. Steatotic Hepatocytes Showed a High Tolerance Towards Oxidative Stress

Oxidative stress is a general feature of NAFLD in vivo [12]. Independent of the initial lipid load, the control groups of PHHs maintained their initial ROS levels for 48 h, a finding confirming that partial hepatectomy and cell isolation increase oxidative stress and antioxidative status [32]. Additionally, we observed that the FFA-treated PHHs and all HLCH showed a significant time-dependent reduction in ROS levels, suggesting a decrease in oxidative stress at a certain lipid load. This finding is in line with recent findings in breast cancer studies, in which lipid accumulation was shown to confer protection against ROS-mediated oxidative stress. It was concluded that lipid droplets are antioxidant and pro-survival organelles that can guard cells against lipotoxic stress [33].

The lower amount of 4-HNE in the FFA-treated compared to the control PPHs indicated reduced lipid peroxidation and suggests an increased cellular protection towards oxidative stress in dependence of the lipid accumulation as well. This effect may be explained by the regulatory function of the nuclear factor erythroid 2-related factor 2 (NRF2) signaling, which is known to limit the progression from NAFLD to NASH by activating genes that promote the elimination of ROS and electrophiles derived from lipid peroxidation and mitochondrial dysfunction [34]. The additional evaluation of oxidative stress using the antioxidant status of GSH did not reveal any differences between the steatotic and control hepatocytes. The initial and early time points of our measurements showed that the GSH concentrations were in line with those of previous observations of isolated human and rat hepatocytes [25,35]. However, after 24 h, the GSH and GSSG concentrations decreased to low levels and GSH depletion had no effect on cell viability. Cassim and coworkers showed that primary mouse hepatocytes displayed a drastic decrease in antioxidative-related metabolites (NADPH, NADP, GSH, and GSSG) during the isolation procedure and subsequent cultivation for up to 48 h compared to those of the in vivo liver. The study demonstrated that GSH depletion to levels below 5% of the control induced cell death only when ATP levels were reduced after the depletion of GSH [35].

To test the sensitivity of the PHHs towards oxidative stress, we treated steatotic hepatocytes with the redox cycling compound menadione. In fact, 10 µM of menadione showed a clear hepatotoxic effect by decreasing membrane integrity and cellular activity. However, menadione incubation did not alter ROS levels significantly. Additionally, PHHs showed a menadione concentration-dependent increase in 4-HNE-protein adducts, which was more pronounced in the control group. Both suggests that additional ROS from redox cycling can remain in balance and can be detoxified and confirms an increased protective capacity towards oxidative stress by steatotic hepatocytes. This finding was confirmed by the GSH concentration, which was increased at 5 µM and decreased at 10 µM menadione treatment in control PHH, indicating an adaptive antioxidant effect at lower oxidative stress levels. These data confirmed the results from Sentellas and coworkers, who showed that a 24 h treatment with 10 µM menadione efficiently reduced intracellular GSH and increased GSSG in rat and human hepatocytes [25]. However, steatotic PHHs showed a later onset of this adaptive increase in GSH concentrations, indicating less sensitivity towards oxidative stress.

### 3.4. Continuous Lipid Accumulation Led to ER Stress

It is known that progression from NAFLD to NASH is accompanied by increasing ER stress in hepatocytes [16]. The ER stress pathways are achieved by IRE1a, PERK, and ATF6, which reduce protein- and lipid-related stress by inhibiting protein translation (PERK), activating autophagy and protein turnover (IRE1a) and β-oxidation (ATF6), and initiating apoptosis (PERK) when regenerative mechanisms fail [36,37]. Therefore, we investigated how the different signaling pathways of ER stress were influenced in a manner dependent on increasing lipid accumulation. Our data showed that the higher lipid load in the HLCH, in general, led to a more pronounced increase in IRE1a, ATF6, and PERK levels. Continuous lipid accumulation in the LLCH and HLCH led to a constant increase in IRE1a indicating an activation of autophagy and protein turnover. In contrast, ATF6 peaked in the LLCH after 72 h and in the HLCH after 96 h, suggesting that a certain threshold of lipid load is reached before ATF6 activation is initiated. ATF6 in turn activates peroxisome proliferator-activated receptor alpha (PPARa) expression and consequently induces mitochondrial ß-oxidation [37]. Therefore, the activation of the ATF6-PPARa axis marks a threshold at which physiological lipid storage starts to turn into lipid stress. The higher threshold of the HLCH may be due to adaptive processes in hepatocytes with chronically elevated lipid load, as observed in the livers of high-fat diet-induced obese mice and in genetically obese ob/ob mice [38].

The induction of lipid stress within the ER is accompanied by an increase in PERK activation. In our experimental setting, the protein amount of PERK showed a general increase with culture time independent of the FFA treatment, probably due to an increasing stress reaction from the artificial 2D culture. However, the change in PERK levels during this culture time was more pronounced in the HLCH cultures suggesting a relation to absolute lipid load. In contrast to steatotic hepatocytes, PERK levels decreased in the control cells in correlation with the decrease in the lipid load. Consequently, PERK peaked in FFA-treated hepatocytes after 72 h in the LLCH and after 96 h in the HLCH, similar to ATF6 activation. Persistent PERK activity leads to the inhibition of protein translation and eventually to apoptosis. However, our data did not show a clear decrease in cell viability or signs of lipotoxicity. Additionally, PERK-activation may also lead to NRF2 nuclear translocation and increased transcription of antioxidant response elements [34]. Our data confirmed that prolonged lipid accumulation in cultured PHH, leading to ER stress, show reduced susceptibility for oxidative stress. Therefore, our data confirmed that TAG accumulation does not induce lipotoxicity per se and could represent a defensive mechanism to reduce excess FFAs, as demonstrated in mouse models [6]. 

### 3.5. Prolonged Activation of the UPR Pathway Decreased Hypatocyte Regenerative Capacity

The activation of the PERK pathway is linked to protein translation inhibition. Therefore, we investigated the impact of lipid accumulation on protein synthesis. It is striking that the HLCH generally showed lower surface protein concentrations than did the LLCH. We previously showed that most surface proteins on PHHs are damaged due to the cell isolation process [39]. The surface proteins and extracellular matrix components are restored during cell culture, as observed in the increasing protein concentrations during the culture period. This regeneration started immediately after seeding the LLCH and reached a maximum at 48 h. However, in the HLCH, only the control PHHs showed delayed regeneration, which was observed after 72 h when the cells had lost a certain amount of stored lipids. The FFA treatment clearly led to a decrease in the protein concentration in the LLCH at later time points and inhibited the regeneration in the HLCH throughout the entire culture time. These data clearly indicate that exceeding a threshold of stored lipids leads to a decrease in hepatic protein synthesis and consequently disturbs cell and tissue regeneration.

In surgery, remaining hepatocytes are likely to proliferate at high levels depending on the protein biosynthesis realized after liver resection. In this process, liver cells carry out increased protein biosynthesis, which in turn causes an increase in protein folding at the ER. This process leads to a rapid increase in ER stress-associated genes such as PERK, pIF2a, and CHOP within the first hours after liver resection [40]. In this regard, the activation of ER stress immediately induces short-term steatosis, which disappears during regeneration [41]. If a non-steatotic liver must stimulate all the necessary processes for regeneration upon resection, then the ratio of induced liver steatosis and activated ER stress must be equally balanced. However, when NAFLD is a preexisting condition of resection, liver regeneration is significantly worse. It is known that advanced NAFLD and NASH increase the risk of liver failure after partial hemihepatectomy [42].

### 3.6. Limitations

Our study is limited by the numbers of donors we investigated. Additionally, the usage of PHHs is accompanied by high individual variances depending on age, sex, disease, lifestyle, and medication. Our data analysis revealed that the initial lipid content has a high impact on the results and that the samples from the six donors had to be separated into PHH groups based on high and low lipid content. However, the analysis of the effects on various interacting pathways confirmed our results with different read-outs, leading to a consistent analysis and revealing a plausible picture. 

Further investigations should address the activation of single ER stress pathways in dependence of prolonged lipid accumulation, because the phosphorylation patterns of ER stress proteins are complex as shown for IRE1a [43]. However, in mice, it was shown that increased levels of total PERK and IRE1a correlated with increased levels of their phosphorylated proteins in dependence of lipid accumulation [44,45].

### 3.7. Prolonged Lipid Accumulation in Primary Human Hepatocytes Led to ER Stress not Oxidative Stress

In summary, our data revealed that hepatocytes are capable of storing enormous amounts of TAG. Lipid accumulation alone did not seem to induce lipotoxicity over the investigated time span. However, FFA-treated PHHs showed signs of different kinds of cell stresses. Although prolonged lipid accumulation did not disrupt redox homeostasis or antioxidative capacity, we observed a lipid load-dependent increase in ER stress. The FFA-dependent increase in ER stress showed thresholds for the activation of pathways associated with lipid stress (ATF6) and inhibition of protein translation and apoptosis (PERK). We concluded that when a certain threshold of lipid-induced ER stress is passed, cellular functions, including regeneration, are impaired. Additionally, persistent PERK activation can induce the activation of apoptosis. In our study, the lipid load was still in a tolerated range, and PERK activation did not lead to cell death.

Our study clearly showed that steatosis per se is a physiological condition and that even severe lipid accumulation can be balanced by cellular defense and regenerative mechanisms. However, fatty liver is also a stressful condition limiting the ability of tissues to deal with additional stress and tissue damage. Tissue damage and regeneration, as it occurs in pathologies, hepatotoxicity, and surgical interventions, intensify the cellular stress that progresses to additional cell loss. Additionally, the regenerative capacities of steatotic hepatocytes are reduced, leading to an increased risk for liver failure. One of the biggest risk factors for liver failure after surgical liver resection is the presence of NAFLD, the incidence of which has dramatically increased in recent years in the Western population. Therefore, this study can help to understand the link between advanced steatosis and impaired cellular regeneration.

## 4. Materials and Methods

### 4.1. Materials, Buffers and Chemicals

Phosphate-buffered saline solution supplemented with calcium and magnesium (PBS) was purchased from Gibco and Trypan Blue was provided by Biochrom (Berlin, Germany). All other chemicals were purchased from Sigma-Aldrich (Munich, Germany), if not stated otherwise. Rat tail collagen was prepared in our laboratory according to the protocol established by Rajan et al. [46].

### 4.2. Tissue Samples

Liver tissue samples were obtained from macroscopically healthy tissue retained after resected human liver in patients with primary or secondary liver tumors or benign local liver diseases (Table 1). All subjects gave their informed consent for inclusion before they participated in the study. The study was conducted in accordance with the Declaration of Helsinki, and the protocol was approved by the Ethics Committee of Charité University Medicine Berlin EA2/076/09 (28 July 2009).

### 4.3. Isolation of Hepatocytes

PHHs were isolated from liver tissue samples by a two-step EDTA/collagenase perfusion technique as described previously [47,48]. The resulting PHH fractions were washed with PBS and re-suspended in PHH culture medium. Afterwards, the cell number and viability were determined in a Neubauer counting chamber by Trypan blue. The cells were seeded on cell culture plastics using hepatocyte culture medium for adherence overnight. PHH culture medium was based on William’s Medium E with GlutaMAX™ (WME, Gibco, Paisley, UK), supplemented with 10% fetal bovine serum (FBS, Merck Biochrom, Berlin, Germany), 15 mM HEPES, 0.1 mM non-essential amino acids (MEM NEAA 100×), 1 mM sodium pyruvate, 100 U/100 µM penicillin/streptomycin (all provided by Gibco, Paisley, UK), 80 IU/l human insulin (Lilly Deutschland GmbH, Bad Homburg, Germany), and 1 µg/mL dexamethasone (Fortecortin^®^, Merck, Darmstadt, Germany).

### 4.4. Use of Hepatocytes in an In Vitro Steatosis Model

In vitro induction of steatosis was achieved on the basis of the model of Gomez-Lechón et al. [31]. After overnight cell adherence, the PHH culture medium was replaced by control or FFA-containing medium (0.6 mM oleic and palmitic acid at a 2:1 ratio in methanol (J.T. Baker, Deventer, Netherlands)). The medium was based on DMEM (PAA Laboratories GmbH, Paschin, Austria) supplemented with 10% FBS, 15 mM HEPES, 0.1 mM MEM NEAA, 1 mM L-Glutamine (Life Technologies, Grand Island, NY, USA), and 100 U/100 µM penicillin/streptomycin. The cell culture medium was changed every 24 h for 5 d. Figure S4 shows the cultivation scheme and the assays performed. Microscopic evaluation was performed with an Axiovert 40 CFL microscope (Carl Zeiss AG, Oberkochen, Germany).

### 4.5. Oil Red O Assay

Oil Red O is a reddishly colored azoic dye used to detect intracellular neutral lipids. Cells were washed once with PBS and fixed with 4% buffered formaldehyde (Herbeta Arzneimittel, Berlin, Germany) for 5 min. For staining, cells were covered in Oil Red O working solution (0.2% of 8.6 M Oil Red O in isopropanol) for 15 min. Afterwards, unbound dye was removed by intensive washing with tap water, followed by a drying phase of at least 20 min. Oil Red O dye was completely dissolved in 100% 2-propanol (Carl Roth GmbH, Karlsruhe, Germany) by gentle rocking. The absorbance was measured at 492 nm using a microplate reader (FLUOstar OPTIMA, BMG Labtech GmbH, Ortenberg, Germany). To calculate relative lipid content the Oil Red O measurement was normalized by the SRB values of the respective sample.

### 4.6. Sulforhodamine B (SRB) Protein Staining

The protein dye SRB binds basic amino acid residues. After the Oil Red O assay, cells were washed twice with PBS and covered with SRB solution for 30 min at room temperature protected from light. Unbound SRB was removed from cells by washing the cultures four times with 1% acetic acid solution. For quantification, SRB was resolved in 10 mM TRIS solution for 10 min. The absorbance was measured at 565 nm using a microplate reader.

### 4.7. XTT Assay

In order to evaluate the cellular and mitochondrial activity as well as changes in energy metabolism, the cell activity was determined using the XTT assay (Roche Diagnostics GmbH, Mannheim, Germany). The assay is based on the reduction of the yellow tetrazolium salt XTT (2,3-bis-(2-methoxy-4-nitro-5-sulfophenyl)-2H-tetrazolium-5-carboxanilide) to a highly colored formazan dye by dehydrogenase enzymes in metabolically active cells. The assay was performed according to the manufacturer’s protocol. After an incubation with XTT reagent of 24 h or 1.5 h for induction of in vitro steatosis or menadione incubation, respectively, the absorbance was measured at a wavelength of 492 nm using a microplate reader.

### 4.8. LDH and AST Assay

To evaluate cellular damage of PHHs, the cell membrane integrity was quantified by measuring the enzymatic activity of LDH and AST in the supernatants using the Fluitest^®^ reaction kits (Analyticon Biotechnologies AG, Lichtenfels, Germany). The assay was performed according to the manufacturers’ instructions. The absorbance was measured at a wavelength of 340 nm at 37 °C using a microplate reader.

### 4.9. DCF Assay

The formation of intracellular ROS was measured by using the fluorogenic substance dichlorodihydrofluorescein diacetate (DCF-DA) according to Kegel et al. [49]. In brief, the cell-permeable DCF-DA diffuses into cells and is deacetylated by cellular esterases and oxidized by ROS to the fluorescent DCF. For ROS measurement, the culture medium was replaced with RPMI medium without serum and phenol red (PAA Laboratories GmbH, Paschin, Austria), but containing 20 µM DCF-DA (Santa Cruz Biotechnology Inc., Heidelberg, Germany) dissolved in DMSO followed by incubation for 30 min at 37 °C, 5% CO_2_ in a humidified incubator. Subsequently, the supernatants were aspirated, and the cells were incubated with RPMI medium for 1 h. Fluorescence was measured in a microplate reader at an excitation wavelength of 492 nm and an emission wavelength of 520 nm.

### 4.10. Glutathion (GSH) Assay

GSH, a ubiquitous tripeptide, and its oxidized form, glutathione disulfide (GSSG) form a redox couple, preventing cellular structures from oxidative damage by neutralizing ROS and are commonly used markers of oxidative stress [50].

Experimental setup followed the protocol of Rahman et al. [51]. Briefly, cells were harvested with trypsin-EDTA (Biochrom AG, Berlin, Germany), washed, and stored resuspended in 30 µL PBS at −20 °C until analysis. The quantification assay was performed in KPE buffer, which was prepared by mixing 16% KH_2_PO_4_ 0.1 M in distilled water and 84% K_2_HPO_4_ 0.1 M in distilled water, adjusted to pH 7.5 or pH 6.1 for GSH or GSSG measurement, respectively, and then adding 8.7 mM EDTA. For quantification, samples were replenished with 220 µL cold extraction buffer (23.6 mM of [0.1% Triton X-100 and 0.6% 5-SSA] in 0.1 M KPE) and lysed by sonification and freeze-thaw cycles.

After centrifugation at 3000 g, 4 °C for 4 min, 100 µL of the supernatant was immediately frozen for later GSH measurements. For GSSG measurement, the pH of another 100 µL of the supernatant was adjusted to 6.1–6.4 and 2 µL of 10% 2-vinylpyridin (Merck, Darmstadt, Germany) in KPE solution was added. After 1 h incubation on ice, cold 16.6% triethanolamine solution was added until a neutral pH was reached. A calibration curve was prepared with different GSH and GSSG concentrations (0.78–50.00 µM). Then, 20 µL of each sample were transferred to a 96-well plate and 100 µL of a solution of 1.33 U/mL glutathione reductase (Roche Diagnostics GmbH, Mannheim, Germany) and 0.84 mM DTNB (5,5′-dithiobis(2-nitrobenzoic acid)) in KPE was added. After 30 s reaction time for the conversion of GSSG to GSH, 50 µL NADPH solution were added, followed by the immediate measurement at 405 nm. The previously frozen GSH samples were thawed. Then, 20 µL of each sample were transferred to a 96-well plate, 0.84 mM DTNB (5,5′-dithiobis(2-nitrobenzoic acid)) in KPE was added, and immediately measured at 405 nm.

### 4.11. 4-Hydroxynonenal (4-HNE) Assay

4-HNE is an α,β-unsaturated hydroxyalkenal that is a product of lipid peroxidation. 4-HNE forms covalent protein adducts, which were quantified by ELISA using the Lipid Peroxidation (4-HNE) Assay Kit (abcam, Berlin, Germany) according to the manufacturers’ instructions.

### 4.12. Induction of Oxidative Stress using Menadione

Menadione, a quinone and potent redox cycler, is a commonly used agent for the study of oxidant-induced liver injury [52,53]. Menadione was dissolved in DMSO and diluted to final concentrations of 0 −10 µM (with a final DMSO concentration of 0.5%). PHHs (two LLCH and two HLCH) were incubated without and with FFA-containing medium as described above for 72 h (Figure S4). Then, the steatotic and control PHHs were incubated with 0, 1, 5, and 10 µM menadione for 24 h and cells were investigated for lipotoxic effects as described above.

### 4.13. Western Blot

For protein analysis, cells were harvested with a cell scraper after 10 min incubation with lysing buffer (0.5 M NaF, 100 mM Na_3_VO_4_, 1 M glycerol phosphate, 4.35% proteinase inhibitor dissolved in (50 mM Trizma^®^ hydrochloride, 0.1% of SDS 10%, 0.5% Triton X-100) in PBS). Protein content was quantified with the bicinchoninic acid (BCA; Interchim, Monteluçon, France) assay according to manufacturer’s protocol. For calibration, a standard series with concentrations from 1000 µg/mL to 62.5 µg/mL was established from a serially diluted solution of 20 mg bovine serum albumin (BSA) in 10 mL distilled water. Samples were adjusted to a protein quantity of 1 µg/µL with water and 5-fold sample buffer and incubated at 96 °C for 5 min prior analysis. For each sample, 20 µg total protein were separated using SDS-PAGE containing 10% acrylamide (Serva Electrophoresis GmbH, Heidelberg, Germany). Protein extracts of MCF-7 cells served as positive control. The electrophoresis was performed at a continuous voltage of 80 V.

The proteins were transferred to a polyvinylidene difluoride (PVDF; EMD Millipore Corporation, Billerica, MA, USA) membrane with a tank blotting system (Mini Trans-Blot^®^ Cell and Module by Bio-Rad Laboratories Inc., Hercules, CA, USA) at a continuous voltage of 30 V at 4 °C for 15–20 h. Primary and secondary antibody solutions were prepared as listed in Table 2. Blocking and incubating solutions were freshly prepared in PBST (0.1% of Tween 20% in PBS) and adjusted to a pH of 7.39–7.41. All washing steps were done with PBST. After blocking at room temperature, incubation with primary antibodies was performed for 15–20 h at 4 °C. The blots were washed five times and incubated for 2 h at room temperature with secondary antibodies coupled with a peroxidase. For the detection of chemiluminescence, the peroxidase and luminol enhancer solutions were mixed in a ratio of 1:1 to incubate the blots for 1 min. Afterwards the blot was placed in a darkroom, where an X-ray film was exposed to light for 10 s to 20 min and later-on processed to an image (Compact 2 X-Ray Film Processor by Protec GmbH & Ko. KG, Oberstenfeld, Germany). Images were scanned by a transparency scanner with a solution of 600 dpi in 16 Bit gray scale values in original size and then analyzed densitometrically with ImageJ 1.46r. Afterwards, the reference protein glyceraldehyde 3-phosphate dehydrogenase (GAPDH) was detected in the same way. All Western blot experiments were done twice independently of each other.

### 4.14. Statistics

Statistical analyses and chart design were performed using GraphPad Prism 7 (San Diego, CA, USA). Data are plotted as the mean values of biological replicates ± standard deviation. Each data point was measured at least in triplicate. For statistical analysis of single values, an unpaired t-test was performed. To test differences among multiple groups, a two-way ANOVA (analysis of variance) was employed, followed by a Dunnett (comparisons of mean values with the control mean value) or Bonferroni (comparison of different treatments) post hoc analysis. A *p* value of 0.05 or less (*) was considered significant, and further significance levels were not differentiated in the figures. All statistical comparisons and significance levels are listed in Table S1, with a representative selection illustrated in the diagrams.

## Figures and Tables

**Table 1:** Donor specifications. List of donor number, age, sex, and diagnosis.

Donor	Age	Sex	Diagnosis
1	70	female	HCC
2	69	male	colorectal liver metastases
3	61	male	NET liver metastases
4	65	male	Klatskin tumor
5	47	male	hemangioma
6	60	male	colorectal liver metastases
7	27	female	hepatocellular adenoma
8	46	female	CCC
9	56	female	hemangioma
10	71	female	CCC

**Table 2:** Antibodies for Western blotting.

Antibody (Manufacturer)	Blocking Time and Solution		Dilution	Incubation Solution
rabbit ATF6α (Santa Cruz Biotechnology Inc., Santa Cruz, CA, USA)	2 h	in 1% BSA	1:200	1% BSA
rabbit IRE1α (Cell Signaling Technology Inc., Danvers, MA, USA)	1.5 h	in 5% milk powder (Carl Roth GmbH, Karlsruhe, Germany)	1:1000	1% milk powder
rabbit PERK (Cell Signaling Technology Inc., Danvers, MA, USA)	2 h	in 1% BSA	1:300	1% BSA
mouse GAPDH (Abcam plc, Cambridge, UK)	1.5 h	in 5% milk powder	1:1000	PBST
donkey anti-rabbit (DIANOVA GmbH, Hamburg, Germany)			1:5000–1:10,000	1% milk powder
donkey anti-mouse (DIANOVA GmbH, Hamburg, Germany)			1:10,000	1% skim milk (AppliChem GmbH, Darmstadt, Germany)

## Supplementary Materials

The following are available online at https://www.mdpi.com/1422-0067/21/19/7097/s1, Figure S1: Increasing lipid content after induction of in vitro steatosis prior to menadione incubation. PHHs (2 × LLCH and 2 × HCLH, donors 7–11) were continuously treated with control medium or 0.6 mM FFA over 72 h. Lipid content was quantified by Oil Red O assay. Data are shown as the means ± SD, *n* = 4 (donors 7–10), two-way ANOVA, and post hoc Dunnett or Bonferroni analysis. A *p* value of 0.05 or less (*) was considered significant; Figure S2: Western blot analysis of the unfolded protein response (UPR) signaling targets (A) IRE1a (130 kDa), (B) ATF6a (90 kDa) and (C) PERK (140 kDa) in the LLCH (donors 1–3) and HLCH (donors 4–6) dependent on FFA treatment. The positive control (+) shows the target signals in MCF-7 cells; Figure S3: Exemplary Western blot analysis of the reference protein GAPDH (36 kDa) in the LLCH (donors 1–3) and HLCH (donors 4–6) dependent on FFA treatment. The positive control (+) shows the target signals in MCF-7 cells; Figure S4: Timeline of PHH cultivation and performed assays; Table S1: Statistical analysis was performed with two-way ANOVA for the main factors time and treatment followed by a Dunnett (comparisons of mean values with the control mean value) or Bonferroni (comparison of different treatments) post hoc analysis. A *p* value of 0.05 or less was considered significant (black values).

## Abbreviations

4-HNE	4-hydroxynonenal
ANOVA	analysis of variance
AST	aspartate transaminase
ATF6a	activating transcription factor 6a
BiP	binding immunoglobulin protein
BCA	bicinchoninic acid
BSA	bovine serum albumin
CCC	cholangiocellular carcinoma
CHOP	CCAAT/enhancer-binding protein homologous protein
DCF-DA	dichlorodihydrofluorescein diacetate
DMSO	dimethyl sulfoxide
ELISA	enzyme-linked immunosorbent assay
ER	endoplasmic reticulum
FFA	free fatty acids
GAPDH	glyceraldehyde 3-phosphate dehydrogenase
GSH	glutathione
GSSG	glutathione disulfide
HCC	hepatocellular carcinoma
HLCH	high-lipid-containing hepatocytes
HSC	hepatic stellate cell
IRE1a	inositol-reqiuring enzyme 1a
LLCH	low-lipid-containing hepatocytes
LDH	lactate dehydrogenase
MEM NEAA	non-essential amino acids
n.d.	not detectable
NAFLD	non-alcoholic fatty liver disease
NASH	non-alcoholic steatohepatitis
NET	neuroendocrine tumor
NRF2	nuclear factor erythroid 2-related factor 2
PBS	phosphate-buffered saline
PBST	PBS with Tween
PERK	protein kinase R-like ER kinase
PHH	primary human hepatocyte
pIF2a	phosphorylated eukaryotic initiation factor 2 alpha
PPARa	peroxisome proliferator-activated receptor alpha
PVDF	polyvinylidene difluoride
ROS	reactive oxygen species
SD	standard deviation
SRB	sulforhodamine B
UPR	unfolded protein response
XTT	2,3-bis-(2-methoxy-4-nitro-5-sulfophenyl)-2H-tetrazolium-5-carboxanilide
