# Supplementary material for: Prolonged Lipid Accumulation in Cultured Primary Human Hepatocytes Rather Leads to ER Stress than Oxidative Stress
Source: Int J Mol Sci. 2020 Sep 26;21(19):7097. doi: 10.3390/ijms21197097 (PMC7582586; doi:10.3390/ijms21197097)
Supplement: Supplementary file 1 [file ijms-21-07097-s001.zip › Supplementary Material.pdf]

International Journal of *Molecular Sciences*

MDPI

Supplementary Materials

Prolonged Lipid Accumulation in Cultured Primary Human Hepatocytes Rather Leads to ER Stress than Oxidative Stress

Christiane Rennert [1,2,†], Theresa Heil [3,†], Gerda Schicht [1,2], Anna Stilkerich [1,2], Lena Seidemann [1], Victoria Kegel-Hübner [1,3], Daniel Seehofer [1,3] and Georg Damm [1,2,3,*]

[1] Department of Hepatobiliary Surgery and Visceral Transplantation, University Hospital, Leipzig University, 04103 Leipzig, Germany; christiane.rennert2@medizin.uni-leipzig.de (C.R); gerda.schicht@sikt.uni-leipzig.de (G.S.); anna.stilkerich@medizin.uni-leipzig.de (A.S.); lena.seidemann@medizin.uni-leipzig.de (L.S.); victoria.kegel@yahoo.de (V.K.-H.); daniel.seehofer@medizin.uni-leipzig.de (D.S.)

[2] Saxonian Incubator for Clinical Translation (SIKT), Leipzig University, 04103 Leipzig, Germany

[3] Department of General-, Visceral- and Transplantation Surgery, Charité University Medicine Berlin, 13353 Berlin, Germany; theresa.heil@outlook.de

* Correspondence: georg.damm@medizin.uni-leipzig.de; Tel.: +49-341-9739656

† Christiane Rennert and Theresa Heil contributed equally as first authors.

Supplementary Figures

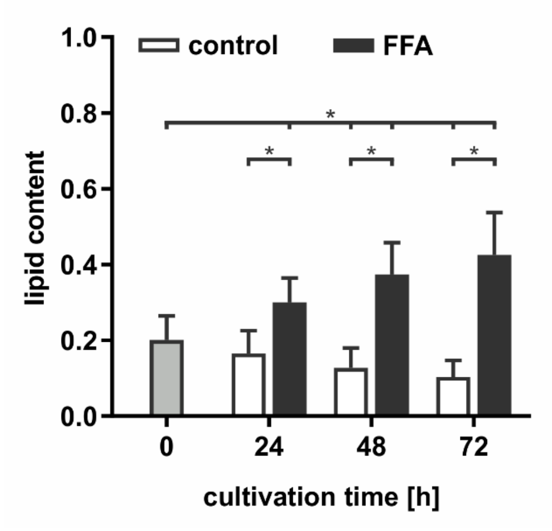

Supplementary Figure S1. Increasing lipid content after induction of in vitro steatosis prior to menadione incubation. PHHs (2 × LLCH and 2 × HCLH, donors 7-11) were continuously treated with control medium or 0.6 mM FFA over 72 h. Lipid content was quantified by Oil Red O assay. Data are shown as the means ± SD, $n = 4$ (donors 7-10), two-way ANOVA, and post hoc Dunnett or Bonferroni analysis. A p value of 0.05 or less (*) was considered significant.

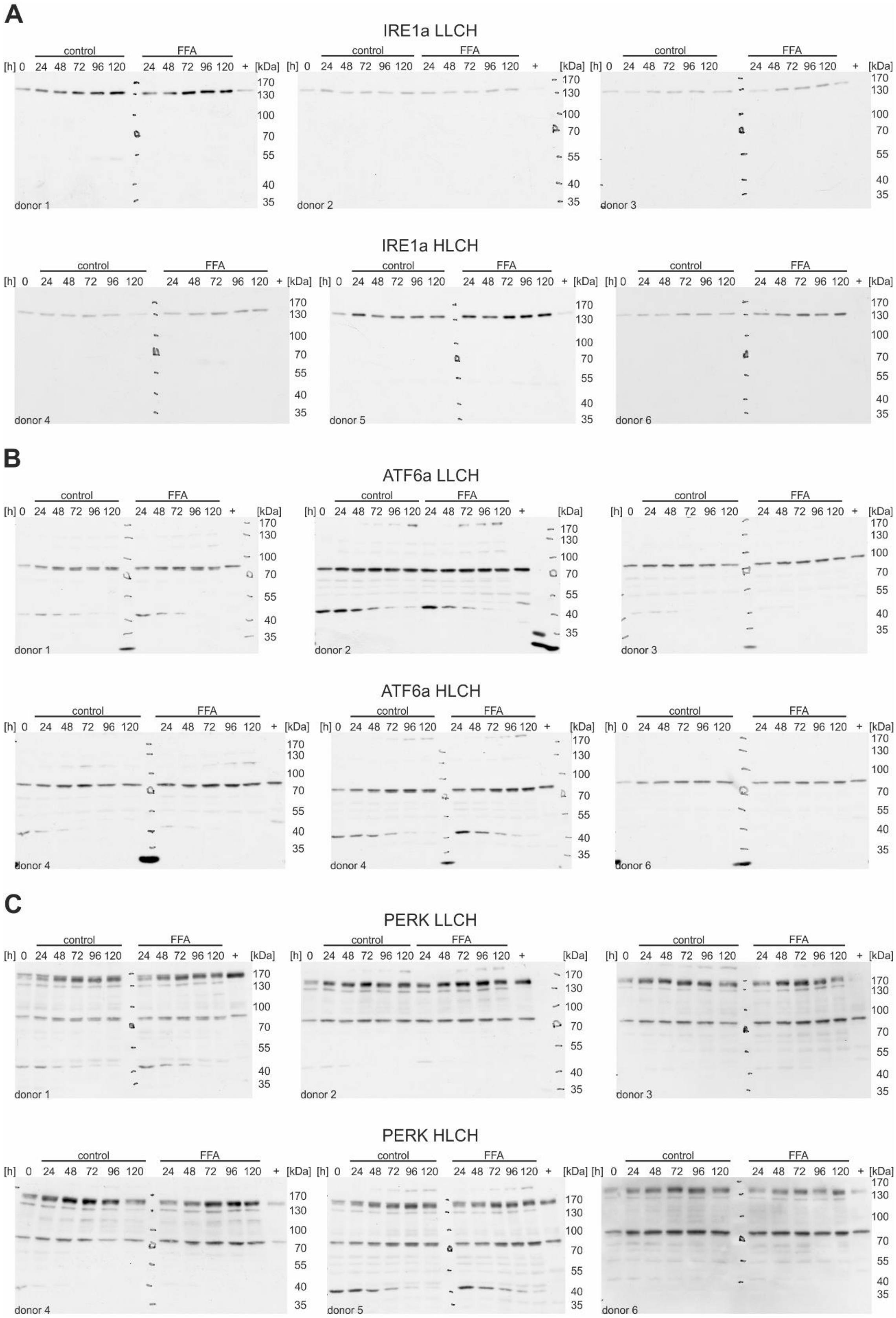

Supplementary Figure S2. Western blot analysis of the unfolded protein response (UPR) signaling targets (**A**) IRE1a (130 kDa), (**B**) ATF6a (90 kDa) and (**C**) PERK (140 kDa) in the LLCH (donors 1-3) and HLCH (donors 4-6) dependent on FFA treatment. The positive control (+) shows the target signals in MCF-7 cells.

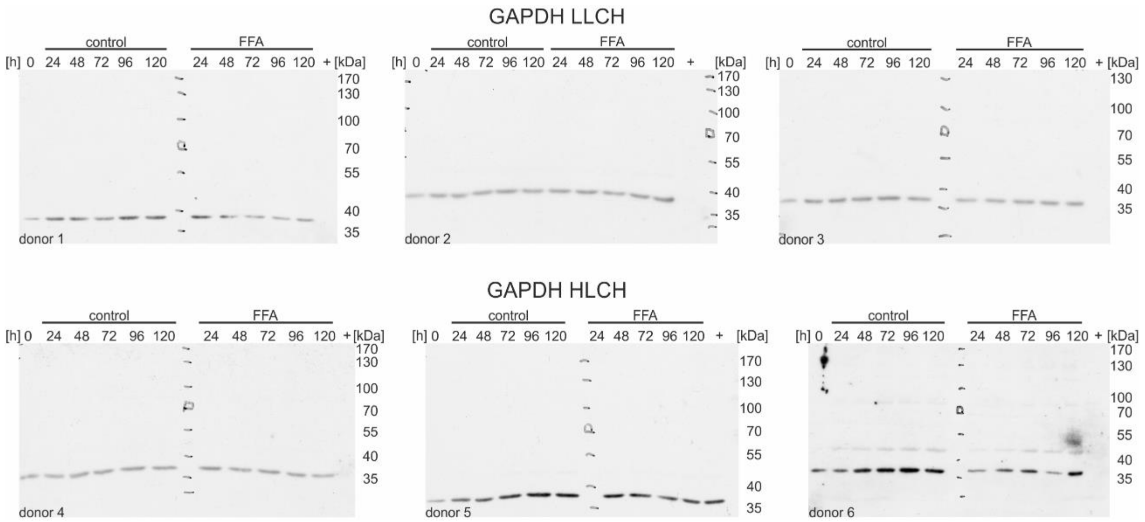

Supplementary Figure S3. Exemplary Western blot analysis of the reference protein GAPDH (36 kDa) in the LLCH (donors 1-3) and HLCH (donors 4-6) dependent on FFA treatment. The positive control (+) shows the target signals in MCF-7 cells.

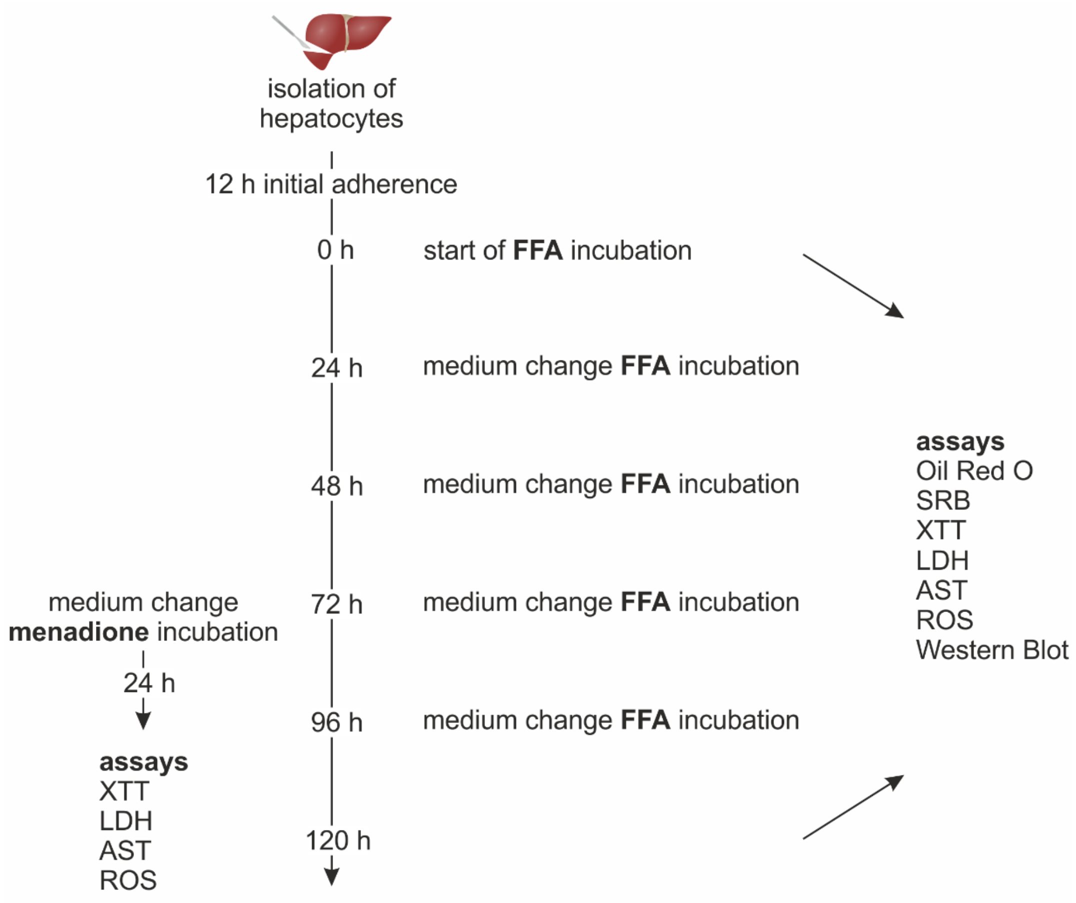

Supplementary Figure S4. Timeline of PHH cultivation and performed assays.

Supplementary Tables

Supplementary Table S1. Statistical analysis was performed with two-way ANOVA for the main factors time and treatment followed by a Dunnett (comparisons of mean values with the control mean value) or Bonferroni (comparison of different treatments) post hoc analysis. A *p* value of 0.05 or less was considered significant (black values).

see separate Excel file 'Supplementary Table S1'
